# Electrochemical Studies of Dithiocarbamates and Related Compounds

**DOI:** 10.6028/jres.093.133

**Published:** 1988-06-01

**Authors:** S. Gomiscek, M. Veber, V. Francetic, R. Durst

**Affiliations:** E. Kardelj University, Department of Chemistry and Chemical Technology, YU-61001 Ljubljana, Yugoslavia; Center for Analytical Chemistry, National Bureau of Standards, Gaithersburg, MD 20899

Dithiocarbamates (DTC) and related compounds are widely used in chemistry, chemical technology, agriculture, and medicine. Therefore, the determination of DTC is of interest not only from a theoretical but also from a practical point of view. Many spectroscopic and electrochemical procedures for their determination have been reported in the literature. Among them, potentiometric monitoring of their concentration seems to be the most promising, in particular if measurements are to be performed in flow systems or with automatic analyzers. As a consequence, there is a demand for a reliable electrode sensor for the DTC^−^ ion.

Some reports have already appeared in the literature [1–3] on DTC ion-selective electrodes using either solid or liquid membranes which enable the DTC^−^ concentration to be determined down to 1×10^−5^ mol/L. Additionally, because of the chelating properties of DTC toward many metal ions, the electrodes are also sensitive to these ions. Therefore, it is not surprising that most of these reports have been concerned more with the potentiometry and potentiometric titration of metal ions than with the determination of DTC^−^ ions using the DTC ion-selective electrode.

The purpose of our work is the development of a sensitive, simple and long-lasting tetramethylene-dithiocarbamate (TMDTC) based electrode sensor which would allow the determination of DTC^−^ as well as metal ions. The dithiocarbamate ion-selective electrode based on the heterogeneous solid membrane (AgTMDTC, Ag_2_S, graphite) was prepared and its performance was evaluated.

## Experimental

The potentiometric measurements were performed using an Iskra MA 5706 pH meter equipped with a saturated calomel reference electrode and the AgTMDTC/Ag_2_S/graphite ISE as the indicator electrode. The polarographic and voltammetric measurements were made using PRG5 Solea-Tacussel and PAR 174 polarographs. All reagents were of A.R. quality, NH_4_TMDTC was prepared in our laboratory, special care being taken to ensure its purity.

## Results and Discussion

**Figure 1:**
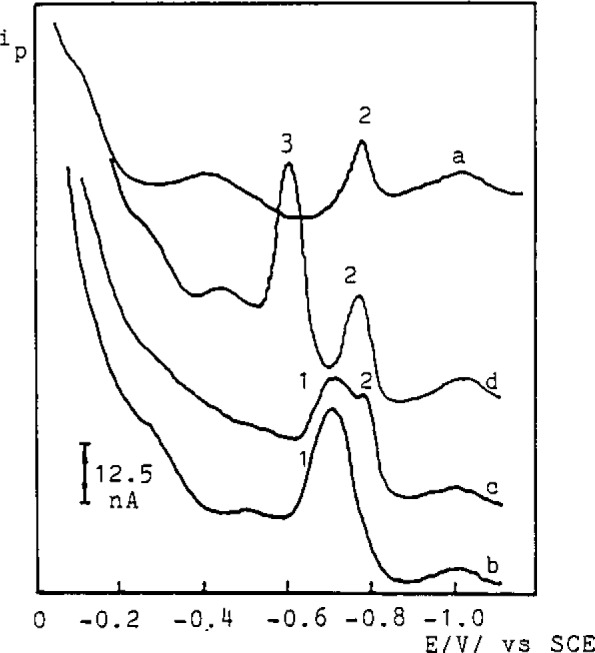
*i-E* curves obtained with differential pulse polarography; a=Cd(TMDTC)_2_ under heterogeneous equilibrium; b = 2.5×10^−6^ mol/L TMDTC^−^; c = 2.5×10^−6^ mol/L TMDTC^−^ +5.0×10^−7^ mol/L Cd^2+^; d = 2.5×10^−6^ mol/L TMDTC^−^ + 5.0×10^−6^ mol/L Cd^2+^. The peaks correspond to TMDTC^−^ (1), Cd(TMDTC)^+^ (2) and Cd^2+^ ions (3).

**Figure 2:**
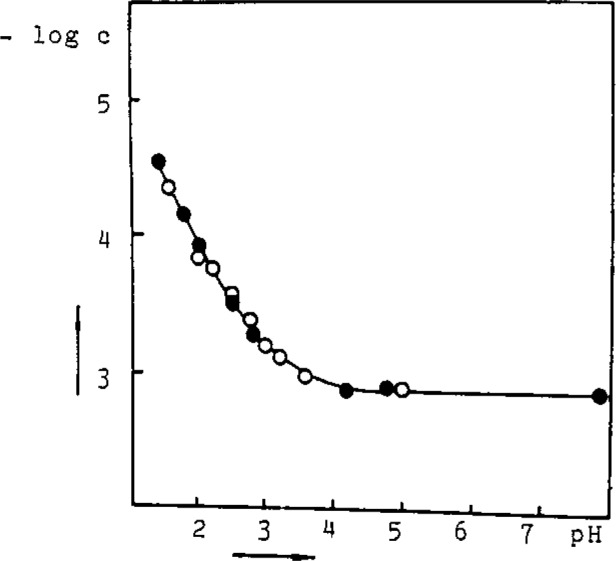
The determination of *K*_a_ with the AgTMDTC/Ag_2_S/graphite ISE; $c_{{\text{NH}}_{4}\text{TMDTC}}=1\times 10^{-3}\text{mol}/L$, 0.1 mol/L KC1 (pH was adjusted with H_2_SO_4_ and NH_3_), O experimental values, • calculated values for p*K*_a_ = 3.1.

Because DTC form slightly soluble complex salts with metal ions in aqueous solutions (*K*_sp_: l×10^−l3^ – l×10^−30^) they can be used in the preparation of membranes for ISE. For this purpose a precipitate of freshly prepared AgTMDTC and Ag_2_S was thoroughly homogenized with a graphite powder (2:1:5), and paraffin oil added to obtain the paste. The electrode shows rectilinear dependence of the potential on the TMDTC^−^ activity/concentration in the range 1×10^−6^ – 5×10^−3^ mol/L. The selectivity of the ISE was estimated for some interfering cations, anions and ligands (table 1). The electrode is useful not only for the determination of TMDTC^−^ and Ag^+^ ion, and the titrimetric determination of metal ions which form slightly soluble complex salts with the TMDTC^−^ ligand but also in the study of chemical equilibria of DTC systems (*K*_sp_, *K*_a_). In addition to the metal ions and DTC^−^ ligand, soluble metal DTC complex salts should not be neglected in the solubility studies. The existence of these species (fig. 1) is clearly indicated by polarographic measurements [4]. The specificity of the AgTMDTC/Ag_2_S/graphite ISE can be used to explain the discrepancies between the *K*_sp_ values reported [5,6] by the determination of the activity of DTC^−^ ions. It was also applied to the determination of the dissociation constants *K*_a_ of DTC acids (Fig. 2) by measurements of the activity of TMDTC^−^ ions at different pH values in a 1×10^−4^ mol/L NH_4_TMDTC solution. The agreement between experimental and calculated values was found to be consistent for p*K*_a_=3.1±0.20 (fig. 2).

## Figures and Tables

**Table 1:** Selectivity coefficients *k*_i,j_ for some interfering ions and ligands

Interfering ion	*C*_i_(mol/L)	*C*_j_(mol/L)	*k*_i,j_
DDTC^−^	10^−4^	5.4×10^−5^	1.84
PO_4_^3−^	10^−5^	3.1×10^−5^	0.32×10^−3^
Cl^−^, Br^−^	10^−5^	N.I.	N.I.
I^−^	10^−5^	4.6×10^−5^	0.22
ETU	10^−5^	7.4×10^−3^	1.35×10^−3^
Urea, ascorbic and tartaric acid	10^−5^	N.I.	N.I.

Selectivity coefficients *k*_i,j_ for some cations		
Cation	*k*_i,j_	log *k*_i,j_
Ag^+^	1	0
Cu^2+^	5×10^−3^	−2.28
Cd^2+^	3×10^−3^	−4.47
Zn^2+^	1×10^−7^	−7.02

N.I.-No interference
